# Evaluation of the add-on NOWAPI^®^ medical device for remote monitoring of compliance to Continuous Positive Airway Pressure and treatment efficacy in obstructive sleep apnea

**DOI:** 10.1186/s12938-016-0139-4

**Published:** 2016-02-27

**Authors:** Damien Leger, Maxime Elbaz, Benoît Piednoir, Amélie Carron, Joëlle Texereau

**Affiliations:** EA 7330 VIFASOM Paris Descartes; Centre du Sommeil et de la Vigilance, Sorbonne Paris Cité, AP-HP Hôtel Dieu de Paris, Université Paris Descartes, 1 place du Parvis Notre Dame, 75181 Paris Cedex 04, France; Air Liquide HealthCare, Medical R&D, Research Centre Paris Saclay, 1, chemin de la Porte des Loges, 78354 Jouy En Josas, France; APHP, Hôpital Cochin, Department of Clinical Physiology, Université Paris Descartes, 27 Faubourg Saint-Jacques, 75014 Paris, France; Air Liquide Applied Maths, Research Centre Paris Saclay, 1, chemin de la Porte des Loges, 78354 Jouy En Josas, France

**Keywords:** Obstructive Sleep Apnea Syndrome, CPAP, Telemetry, Remote Monitoring, Compliance

## Abstract

**Background:**

Optimizing the measurement of Continuous Positive Airway Pressure (CPAP) compliance and treatment efficacy is paramount for patients with obstructive sleep apnea syndrome (OSAS). Compliance knowledge is currently based on data coming from CPAP machines; however algorithms and measured parameters vary from one machine to another. This study was conducted to clinically evaluate a novel device, NOWAPI^®^, designed to assess compliance remotely in conjunction with any CPAP machine. NOWAPI^®^ was tested against polygraphy, the gold standard for the measurement of CPAP treatment duration and residual apnea-hypopnea index (AHI).

**Methods:**

Single group assignment, open label, non-randomized. Sleep laboratory setting. 22 adult patients with OSAS treated by CPAP were included. Recordings were performed during one night while the patient was treated with his/her usual CPAP and interface. NOWAPI^®^ data were collected electronically and compared to data acquisition and visual scoring using an EMBLETTA^®^ GOLD polygraph. Statistics were only descriptive.

**Results:**

Recordings were performed with six different CPAP machines and three different interfaces (full facemask, nasal pillow, nasal mask). The median [Q1; Q3] absolute difference in CPAP treatment duration between NOWAPI^®^ and polygraphy was of 1.0 min [0.0; 12.0], corresponding to a relative difference of 0.21 % [0.0; 2.2] (Per Protocol data set, n = 20). NOWAPI^®^ tended to underestimate residual AHI in a magnitude of two events per hour as compared to polygraphy. The device was well tolerated and the patient satisfaction was good.

**Conclusions:**

This clinical study confirmed prior bench tests, showing that NOWAPI^®^ estimate of CPAP treatment duration was clinically acceptable and in agreement with polygraphy. Although a limited number of OSAS patients treated by CPAP were included, relevant findings for the device improvement were identified.

*Trial Registration* ClinicalTrials.gov identifier: NCT01441622. The study was funded by Air Liquide HealthCare

## Background

Sleep apnea syndrome (OSAS) has a high prevalence and has serious consequences on public health with high costs for diagnosis and treatments [1–3]. It is associated with an increased risk of accidents and cardiovascular comorbidities [4, 5]. The standard treatment for OSAS is Continuous Positive Airway Pressure (CPAP) which improves the quality of sleep and alertness and decreases the associated risks. A minimum of 3–4 h of treatment every night is necessary to obtain long term benefit [6, 7]. In several European countries, this minimum compliance has been proposed as a “cut-off” minimum limit per night by payers for reimbursement of CPAP [2, 7]. Furthermore, remote monitoring was shown beneficial to CPAP compliance [8].

**Fig. 1:**
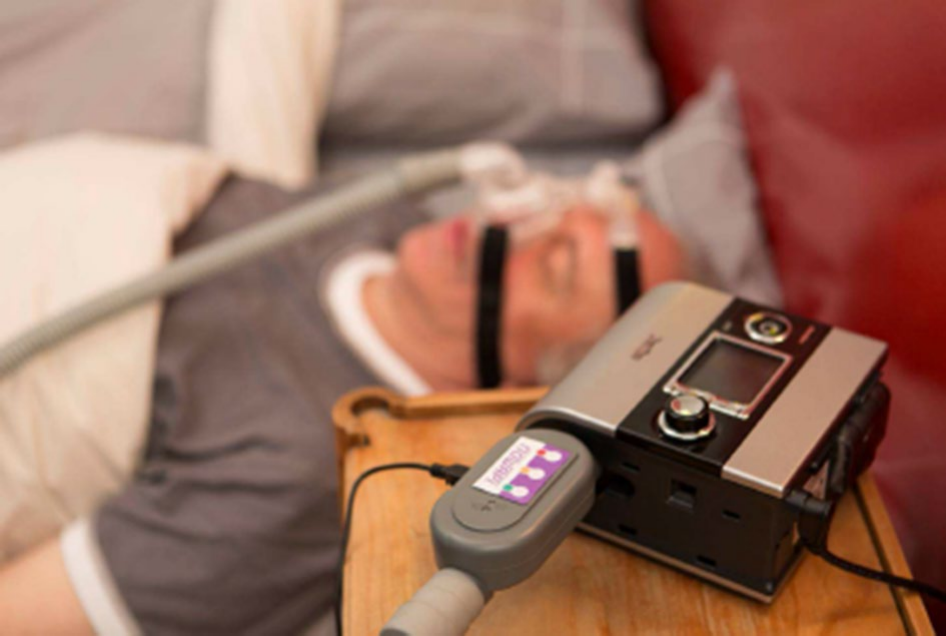
NOWAPI^®^ stands on the patient circuit, at the output of the CPAP device. The diode indicator gives a traffic light feedback to the patient on the previous night CPAP treatment duration

**Fig. 2:**
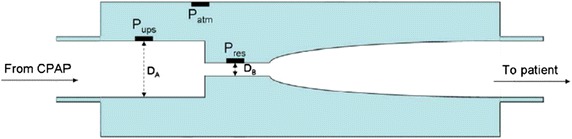
Schematic view of the NOWAPI^®^. *D*_*A*_ *and D*_*B*_ diameter upstream and at restriction site, respectively. *P*_*atm*_ atmospheric pressure sensor, *P*_*ups*_ pressure sensor upstream of the restriction, *P*_*res*_ pressure sensor at restriction site

Compliance is currently assessed on CPAP outputs with algorithms varying from one model to the other such that there is no standardization of data. The NOWAPI^®^ medical device (Fig. 1) has been designed to remotely monitor duration of CPAP use, residual number of apneas and hypopneas, leaks and delivered pressure, whatever the machine used. The device is connected to the breathing circuit at the CPAP output. It uses a tunnel with area changes for measuring instantaneous pressure and flow rate inside the patient circuit (Fig. 2). The technology is especially designed for the detection of precise flow variations and the identification of breathing cycles. The device was shaped to detect these variations without affecting the treatment (very low pressure losses). Identification of apneas and hypopneas is based on consensual definitions: an apnea is defined as a 10 s event with a tidal volume less than 20 % of the patient reference tidal volume; a hypopnea is defined as a 10 s event with a tidal volume between 20 and 50 % of the patient reference tidal volume. The device can store 1 month of data in the absence of electrical power, and up to 1 year if plugged in. The information can then be transmitted by GSM/GPRS on a daily basis or recovered by connecting the device directly to a computer.

The device was previously validated in a bench test simulating OSA patients [9]. Tests concluded that NOWAPI^®^ did not influence the CPAP treatment (no pressure drop) and presented a good performance for detecting the treatment duration (never higher than 3 min over a 4-hr test) and residual events (no significant differences in AHI estimates against CPAPs). This clinical study was conducted to further evaluate the performance of the NOWAPI^®^ in closest to real conditions of use, against independent measurements during lab polygraphy. This exploratory study was designed as solely descriptive and included a sample of 22 patients treated for OSAS.

## Methods

### Study design

The study was single centre, one group, non randomised open exploratory study. Eligible patients were adults with predominantly obstructive OSAS (>50 % of obstructive events), meeting CPAP treatment criteria, treated for at least 2 months and requiring an in-hospital night respiratory polygraphic recording. It was planned to enroll at least 10 patients or more with a residual AHI above 10 per hour of sleep, based on the last home provider report available. Patients were non eligible if they had chronic respiratory disease, acute rhinitis, rhinopharyngitis, moderate to severe chronic heart failure, Cheyne-Stokes respiration, not under control progressive illness which could interfere with study procedures.

The study protocol was approved by an Independent local Ethics Committee and by the French Drug Agency. The study was performed in accordance with Good Clinical Practice guidelines. All patients gave their informed consent before initiation of any measures specific to the study.

In order to test the NOWAPI^®^ performance in various and random situations, evaluations were performed in usual conditions of a nocturnal respiratory polygraphy recording in the sleep lab, with the usual patient’s equipment including his CPAP device, interface and in some cases humidifier. Patients were equipped in the evening and were given a sleep agenda and a satisfaction questionnaire. In the morning, the equipment was removed and questionnaires were collected. All events which occurred overnight that might have interfered with the recordings were reported.

### Data collection

Duration of CPAP treatment, number of apneas and hypopneas and mean positive pressure measured by NOWAPI^®^ were compared to those measured by respiratory polygraphy (Embletta GOLD^®^, ResMed), routinely used for the diagnosis and severity evaluation of OSAS conjointly with clinical sleep evaluation [10].

The two different files the NOWAPI^®^ stores were collected: the summary data file of mean values recorded by consecutive 15-min periods and the detailed data file including the data recorded every 40 ms.

The investigator estimated CPAP treatment duration and the numbers of apneas and hypopneas on the same 15-min recording periods as the NOWAPI^®^ during the recording night and reported them on the Case Report Form. Visual scoring of apneas and hypopneas from respiratory polygraphic data was performed according to AASM 2007 guidelines [11].

Patient opinion on NOWAPI^®^ was evaluated through a self-administered questionnaire. It was divided in two parts: the general aspect of the device (three questions) and the light-emitting diode (two questions). Answers were given using a satisfaction scale (0, 20, 40, 60, 80, 100 %), 0 % being the poorest satisfaction and 100 % the maximum.

Sleep was evaluated by the patient through a sleep-log, which was used to calculate subjective total sleep time (TST), sleep onset latency (SOL), wake after sleep onset (WASO) and time in bed (TIB).

### Statistical analysis

All statistical results of this study were exploratory and exclusively descriptive. This study was purely exploratory and a pragmatic approach had been chosen to estimate its sample size. As no assumption could be made prior to the start of the study on the primary efficacy criterion, no sample size calculation could be performed. Thus, the number of evaluable patients chosen was 20. This was expected to be sufficient to establish preliminary descriptive conclusions regarding NOWAPI^®^ performance in estimating the duration of CPAP treatment. An “evaluable” patient was a patient with a record duration ≥240 min for both NOWAPI^®^ and polygraph.

The primary analysis consisted in assessing the absolute difference in CPAP treatment durations estimated by NOWAPI^®^ and by respiratory polygraphy, in the Per Protocol Set (PPS), i.e. all evaluable patients. The CPAP treatment duration estimated by the NOWAPI^®^ during the recording night was obtained by summing all CPAP treatment durations estimated during all 15-min recording periods of the recording night. The overnight CPAP treatment duration was selected as the primary variable rather than the 15-min values as this is the quantitative indicator of patient observance communicated to the physician.

The mean of absolute differences (absolute value of differences) between the number of apnoeas and hypopneas estimated by the NOWAPI^®^ and estimated by the investigator from polygraphic data were calculated for each of the 15-min periods included in the recording night and overall (over all the 15-min periods of recording included in the recording night). The 15-min values are more of interest from an engineering point of view to determine the accuracy of NOWAPI^®^ whether the overall value is a quantitative indicator of CPAP treatment quality communicated to the physician.

A post hoc analysis was performed on a patient subgroup for which detailed NOWAPI^®^ data files were available.

Statistical analyses were performed using SAS^®^ Software, version 9.2. (SAS Institute, North Carolina, USA).

## Results

Patients were mainly men (18/22), aged 61 ± 13 years and with BMI of 29.6 ± 4.5 kg/m^2^ (mean ± SD). Their baseline characteristics are outlined in Table 1. Reasons for the in-hospital night respiratory polygraphic recording were: unrefreshing sleep (10/22), recurrent awakenings (8/22), disturbed sleep (5/22), excessive daytime sleepiness (5/22), poor compliance to CPAP treatment (2/22) and residual AHI >20/h (1/22). From available prior home CPAP reports (n = 21), residual AHI was 5.8 ± 5.5/hr and in only three patients was ≥10/hr, whereas 10 such patients were requested per protocol. Two patients with major protocol deviations (less than 4 h of respiratory polygraphic recordings, i.e. 0 and 2.25 h respectively) were not included in the main analysis. Interfaces included 12 face masks, 8 nasal masks and 2 nasal pillows. 20 patients had an auto-adjusting CPAP device and two had a conventional CPAP device. 6 different CPAP devices were used [S8 AutoSet Spirit™ II (ResMed), S9 AutoSet™ (ResMed), REMstar Auto A-Flex™ PR One (Philips Respironics), REMstar Auto M Series with A-Flex (Philips Respironics), GoodKnight^®^ 420 (Tyco healthcare), KXS-Bump (Kaerys)]. Fourteen patients had a humidifier and eight none. Four patients rose from bed and three patients removed their interface during the recording night.

**Table 1:** Characteristics of the study population (full analysis set, n = 22)

Age (years)	61 ± 13
Gender (% male)	82 %
Body mass index (kg/m^2^)	29.6 ± 4.48
Neck circumference (cm)	40.6 ± 3.8
Waist circumference (cm)	106.5 ± 13.5
Time since OSAS diagnosis (years)	3.2 [0.5–5.3]
Epworth scale score at diagnosis	11.8 ± 4.0
Polygraphic data at diagnosis	
Apnoea-hypopnoea index (/hr)	43.0 ± 16.6
Number of obstructive events (/hr)	28.9 ± 16.9
Mean nightly CPAP use (hrs) in the month prior selection	5.5 ± 2.2

Time since OSAS diagnosis is expressed in median [Q1–Q3]. All other values are normally distributed and expressed as mean ± SD

*OSAS* obstructive sleep apnea syndrome, *CPAP* continuous positive airway pressure, *Q1* first quartile, *Q3* third quartile

**Fig. 3:**
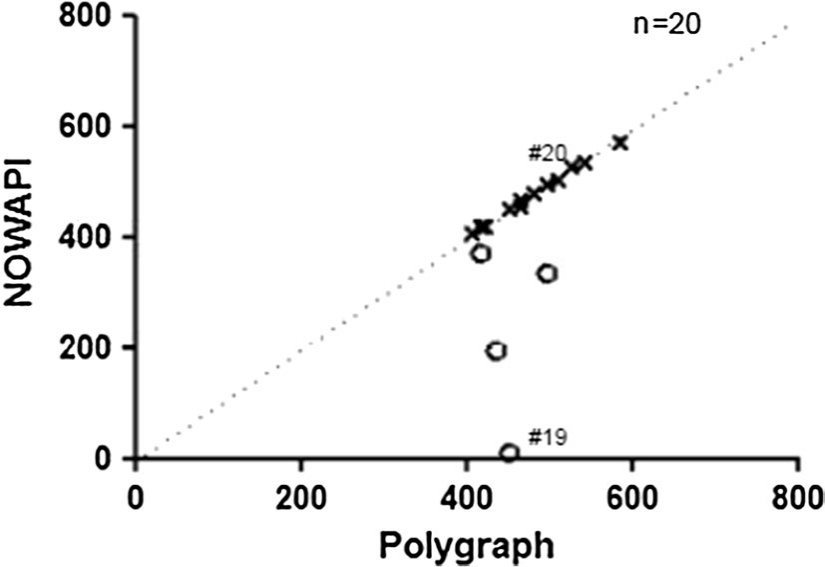
Comparison of overnight CPAP treatment duration (minutes)—Respiratory Polygraph vs NOWAPI^®^ (PP data set, n = 20).* Crosses* represent the 16 patients with a perfect agreement in CPAP treatment duration measurement between NOWAPI^®^ and polygraphy, all crosses being aligned on the 1:1 fit line. The circles represent the four outliers. Detailed NOWAPI^®^ data files of patients #19 and 20 are presented in Fig. 4

Considering the overall recording night, the median CPAP treatment duration estimated by NOWAPI^®^ and from polygraphy was of 471 min (ranging from 11 to 570) and 479 min (ranging from 405 to 585), respectively (PPS). The median [Q1; Q3] absolute difference in CPAP treatment duration between NOWAPI^®^ and polygraphy was of 1.0 min [0.0; 12.0], corresponding to a relative absolute difference of 0.21 % [0.0; 2.2] (PPS). The maximum absolute difference was of 439 min (97.6 % in relative absolute difference). Figure 3 presents for each patient the CPAP treatment duration measured by NOWAPI^®^ against the one measured by polygraphy. It shows that NOWAPI^®^ measured the exact same treatment duration as polygraphy in all but four patients.

**Fig. 4:**
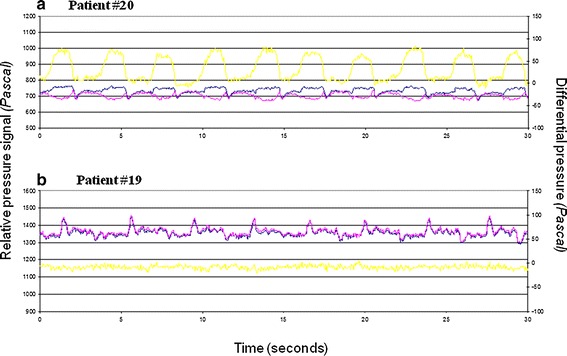
Example of NOWAPI^®^ detailed data in two typical patients. The device measures two pressure signals (upstream restriction and at the site of restriction, *pink and blue curves, left y*-*axis*). Variations in the difference between two pressures (differential pressure, *yellow curve, right y*-*axis*) indicate a flow rate is detected. *Panel* **a** shows detailed data in patient #20, with variation in differential pressure indicating detection of a flow rate in the patient circuit, i.e. a CPAP treatment. *Panel* **b** shows detailed data in patient #19. The two pressure signals are always superimposed, there is no variation in differential pressure. This finding is compatible only with two situations, either no flow rate in the patient circuit, either reverse connection

Examination of NOWAPI^®^ detailed data files was conducted to further analyze the CPAP treatment duration results and in particular the outliers. Detailed data files were available in only 18 of the 20 patients of the PPS. An example of NOWAPI^®^ detailed data is shown in Fig. 4: flow rate determination (i.e. treatment detection) is allowed by calculating the difference between the two pressure signals (pink and blue curves). If this difference (yellow curve) varies, it means that there is a flow rate, like what is seen in patient #20. In patient #19, the two pressure signals are always superimposed: this finding is compatible only with two situations, either no flow rate in the patient circuit, either reverse connection of the NOWAPI^®^ on the patient circuit. Review of the 18 NOWAPI^®^ detailed data files showed no variation in differential pressure (indicating with high probability the reverse connection of the NOWAPI^®^ on the patient circuit, gas flow in the wrong sense through the device, during the recording night) in the 4 outlier patients. NOWAPI^®^detailed data files of all other 14 patients showed the expected variation in differential pressure.

A post hoc analysis was therefore conducted on the 14 patients having a detailed data file indicating correct connection: Considering the overall recording night, the median CPAP treatment duration estimated by NOWAPI^®^ and from respiratory polygraphy was of 478 min (ranging from 405 to 570) and 479 min (ranging from 405 to 585), respectively. Median [Q1; Q3] absolute difference in CPAP treatment duration between NOWAPI^®^ and respiratory polygraphy was of 0.0 min [0.0; 1.0], corresponding to a relative difference of 0.0 % [0.0; 0.24] overall on the recording night for this subgroup. The maximum absolute difference was of 15 min (2.6 % in relative difference).

**Fig. 5:**
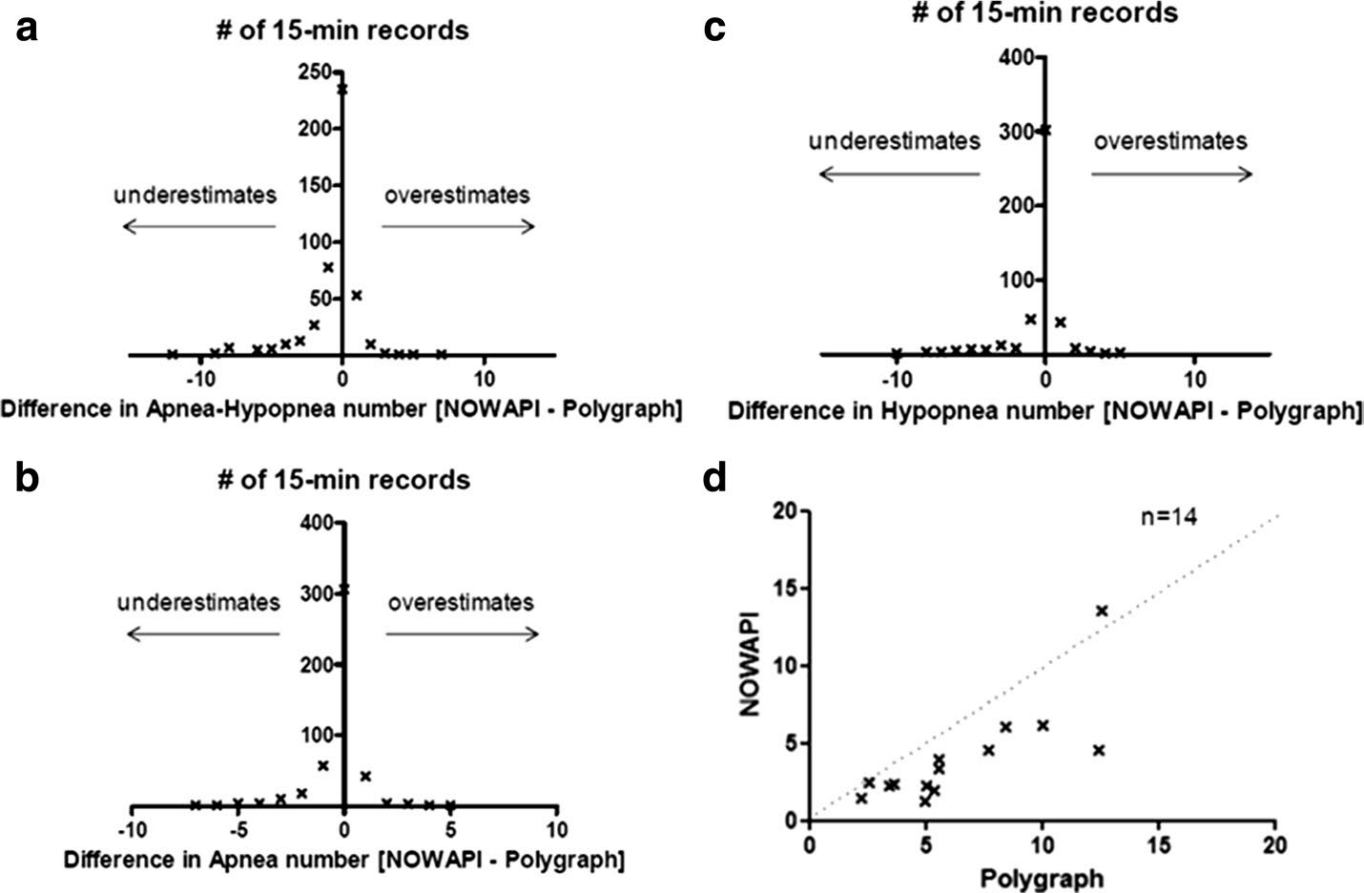
Differences between numbers of apneas and hypopnoeas (*Panel* **a**), apneas (*Panel* **b**) and hypopneas (*Panel* **c**) estimated by NOWAPI^®^ and by respiratory polygraphy are presented for each of the common 15-min periods (n = 452) of the recording night. The *x-axis* plots the difference [NOWAPI^®^ minus Respiratory Polygraph] and the *y*-*axis* the number of records. *Panel* **d** presents the Apnea-Hypopnea Index (events per hour) by patient for the overall recording night, estimated by NOWAPI^®^ plotted against polygraphy with a 1:1 fit line. PP data set with detailed NOWAPI^®^ data files indicating correct connection (n = 14)

Evaluation of the performance of the NOWAPI^®^ in apnea and hypopnea detection was therefore conducted only in patients for whom NOWAPI^®^ detailed data file examination indicated a correct device connection. Differences between numbers of apneas and hypopneas estimated by NOWAPI^®^ and from polygraphic data interpretation are presented for each of the common 15-min recordings in Fig. 5 (panels A, B and C). Agreement between NOWAPI^®^ and polygraphy was within two apneas or less in most records (427/452, i.e. 94 %), and similarly within two hypopneas or less in most records (408/452, i.e. 90 %). Underestimation of more than two respiratory events par NOWAPI^®^ (20/452 and 37/452 of records in apnea and hypopnea detection respectively) was most frequent than overestimation of more than two respiratory events (5/452 and 7/452 of records in apnea and hypopnea detection respectively). Considering the overall recording night, the median [Q1; Q3] absolute difference in AHI was 2.2 [1.1; 3.4] events per hour between NOWAPI^®^ and polygraphy. Figure 5 (Panel D) shows residual AHIs measured by NOWAPI^®^ plotted against respiratory polygraphic data interpretation for the 14 patients overall on the recording night, and confirmed NOWAPI^®^ underestimation of residual respiratory events, in an order of magnitude of about two events per hour.

From the sleep agenda, TST was 433.0 ± 105.8 min (around 7.2 h), SOL was 32.8 ± 27.8 min, WASO was 34.4 ± 49.5 min and TIB was 501.9 ± 51.2 min (around 8.3 h) (n = 22) (mean ± SD).

**Fig. 6:**
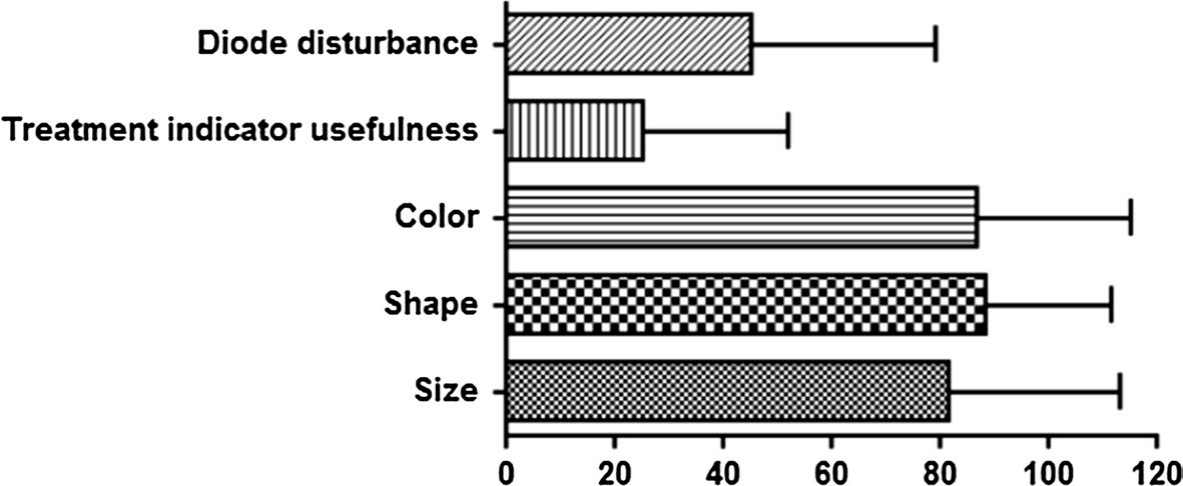
Patients’ opinion on NOWAPI^®^ (n = 12). Results are mean ± SD. The self-administered satisfaction questionnaire included three questions on the general aspect of the device and two questions on the light indicator. Answers were given using a satisfaction scale (0, 20, 40, 60, 80, 100 %), 0 % being the poorest satisfaction and 100 % the maximum

Figure 6 shows patient opinion on NOWAPI^®^. Patients were globally highly satisfied of the design of the device (shape, size, color). Diode indicator, included to provide feed-back to the patient on his compliance, obtained the lowest satisfaction rating. No patient reported that the traffic light indicator was very helpful (100 % rating) in indicating the quality of the treatment and 42 % of patients reported that it was not helpful at all (0 % rating). Conversely, the patients quite high-rated disturbance during the night, 8 % of patients having reported high disturbance (100 % rating) and 17 % of patients reported no disturbance (0 % rating).

No adverse event was reported during the study.

## Discussion

Our study shows that, when correctly connected, NOWAPI^®^ can reliably measure CPAP treatment duration versus respiratory polygraphy, independently of the mask or the CPAP applied, either conventional or auto-CPAPs. Following the bench tests, the study aimed to evaluate the clinical feasibility and accuracy of this new device in a small group of patients treated by different CPAPs and interfaces. We used respiratory polygraphy as a reference to assess NOWAPI^®^ reliability as recommended in consensus guidelines for the diagnosis and monitoring of OSAS in adults [10]. Respiratory polygraphy is indeed regularly used under the supervision of qualified sleep technicians for the follow-up of patients with OSAS who have been diagnosed and treated for several months.

Treatment duration is a key parameter for the long-term assessment of patients with OSAS treated at home by CPAP, because duration is strongly associated with effectiveness of CPAP therapy. NOWAPI^®^ defines a treatment period by a period during which both a positive pressure and a patient breathing are detected. Most of the current CPAP devices accurately measure CPAP treatment duration, as it is the operating time of the devices. From respiratory polygraphic data, the investigator was also able to estimate accurately the CPAP treatment duration, although this is not a usual assessment, especially in specific predetermined 15-min periods. The results of this clinical study provided a necessary further assessment of the NOWAPI^®^ performance than the one provided by the bench tests. The bench test [9] indicated a difference in treatment duration never higher than 3 min (1.25 % in percentage time difference) and never lower than 1 min (−0.42 % in percentage time difference). Our study showed that NOWAPI^®^ measured the exact same CPAP treatment duration than polygraphy in 16/20 patients and underestimated the duration in the remaining 4, sometimes remarkably (almost no treatment detection in one patient) (Fig. 3). In those four patients, analysis of the NOWAPI^®^ detailed data files indicated reverse connection of the NOWAPI^®^ during the recording night, a possibility that could not have been detected by a bench test and was not anticipated because NOWAPI^®^ has two different ends (one male and one female). It is of important note that even with a misconnected NOWAPI^®^, the positive pressure was properly administered because the NOWAPI^®^ measures flow not using a pneumotachograph but with a customized design that minimizes pressure drops (Fig. 2).

Residual (i.e. under CPAP treatment) AHI is also an important parameter to monitor in patients with OSAS because it is an indicator of quality of the treatment: an AHI lower than 10 should be achieved with appropriate CPAP settings and interface. The bench test [9] indicated an AHI difference of 0.9 ± 1.6 events per hour between NOWAPI^®^ and the bench actual values. The clinical study showed that agreement between NOWAPI^®^ and respiratory polygraphy was within two apneas or less in 94 % of all 15-min records (Fig. 5b) and within two hypopneas or less in 90 % of all 15-min records (Fig. 5c). When summing all differences over the recording night, it appeared that NOWAPI^®^ mainly underestimated AHI (in 12/14 patients, Fig. 5d), the median absolute difference being of 2.2 events per hour. Having a difference in apnea and hypopnea scoring between NOWAPI^®^ and respiratory polygraphic interpretation was expected, as the scoring methods differ. NOWAPI^®^ defines an apnea with a decrease of 80 % or more of the tidal volume that lasts at least 10 s and an hypopnea if there is a decrease of 50 % or more of the tidal volume that lasts at least 10 s. According to published guidelines for respiratory polygraphic interpretation [11], (i) an apnea is scored when there is a reduction of at least 90 % of the flow signal from baseline, lasting at least 10 s (the decrease in flow should be present during at least 90 % of the total event duration) and (ii) a hypopnea is scored when there is a reduction of at least 30 % of the flow signal from baseline, lasting at least 10 s (the decrease in flow should be present during at least 90 % of the total event duration), and associated with an arterial desaturation of 3 % or more. However, main objective of the long-term home monitoring of CPAP treatment is not to precisely measure AHI but to detect the patients with high residual event rates, i.e. those requiring a CPAP treatment adjustment (settings, interface,…). One main study result is that AHI underestimation by NOWAPI^®^ would have resulted in missing to detect two patients with residual AHI ≥10/hr (with polygraph) and requiring treatment adjustment.

Patient satisfaction regarding the general aspect of the device was good (Fig. 6). However, low satisfaction regarding the traffic-light indicator (helpfulness and light disturbance) was not expected. It will require deeper understanding of the patients’ needs and wishes in term of CPAP treatment feedback.

The main limitation of the present study is the small number of patients. However, the study was exploratory and was not designed to demonstrate statistical equivalence with ventilatory polygraphy. Sample size was not estimated and 20 evaluable patients were anticipated enough for this pilot study of first use in OSAS patients. Indeed, even with the limited number of patients, the study allowed to identify that misconnection was possible despite the two different NOWAPI^®^ ends, a useful information before a wider use. Second, the study allowed testing the novel device with six frequently used CPAP machines, the three available interfaces and with/without humidifier. Third, the study showed that NOWAPI^®^ tended to underestimate AHI, and this was not identified by the bench tests. Underestimation might not be the best option if the main aim of remote monitoring is to detect patients with high residual events in which CPAP treatment needs adjustment. Another limitation is that although it was planned to include 10 patients with a residual AHI of 10 or above, only three were included. This limits the conclusions that can be made on the NOWAPI^®^ performance in detecting patients with high residual events, but reflects the real life situation, i.e. that very few patients have a high residual AHI under CPAP treatment.

## Conclusion

The aim of the present study was to clinically evaluate a novel add-on device designed to monitor continuously patient compliance and CPAP treatment efficacy in OSAS patients. The device accuracy was tested against respiratory polygraphy. The results of this study suggest that, when correctly connected, NOWAPI^®^ allows a precise measurement of CPAP treatment duration, the main parameter to be monitored on the long-term to ensure a proper patient follow-up and implementation of adequate patient support in case compliance is decreasing.

Although a limited number of patients was included, the study has shown three relevant concerns that were not identified in the bench test and should be corrected in the perspective of a larger use at patient homes:

- First, reverse connection of the device on the patient circuit was possible despite different ends. Although reverse connection did not raise any safety concern (CPAP treatment was normally delivered and no adverse event occurred), duration of CPAP treatment was in this case inaccurate. A clearer indication of the flow direction is to be implemented on the device.
- Second, AHIs estimated by NOWAPI^®^ slightly differed from the ones obtained by interpretation of respiratory polygraphic data, resulting in a AHI underestimation of about two events per hour. This finding was expected by the difference in the methods used to score respiratory events. However, this may lead to miss some patients requiring a treatment adjustment and will require a modification of the NOWAPI^®^ scoring algorithm.
- Third, low satisfaction rate of the patients about the traffic-light indicator suggests that other means to provide feed-back to the patient about compliance and treatment efficacy are to be considered and the need for a specific focus on CPAP treatment education by the home healthcare provider.

OSAS is a common condition associated with potentially severe outcomes, to which a chronic disease management model should be applied. NOWAPI^®^ could be a useful tool to monitor in the early and long term treatment compliance and quality. Because patients are treated with many types of CPAPs and interfaces, and because each CPAP machine has its own event detection algorithm and report [12], adding-on a monitoring device such as NOWAPI^®^ would allow provision of standardized measures and reports and it should be i.e. easier to compare a patient to himself when he had tried different CPAP machines.

## Abbreviations

AASM: American Academy of Sleep Medicine
AHI: apnea-Hypopnea Index
95 % CI: 95 % Confidence Intervals
CPAP: Continuous Positive Airway Pressure
GSM/GPRS: global System for Mobile communications/General Packet Radio Service
OSAS: obstructive sleep apnea syndrome
SD: standard Deviation
SOL: sleep Onset Latency
TIB: time In Bed
TST: total Sleep Time
WASO: wake After Sleep Onset
